# Development and Co-design of NeuroOrb: A Novel “Serious Gaming” System Targeting Cognitive Impairment in Parkinson’s Disease

**DOI:** 10.3389/fnagi.2022.728212

**Published:** 2022-03-29

**Authors:** Bianca Guglietti, David A. Hobbs, Bradley Wesson, Benjamin Ellul, Angus McNamara, Simon Drum, Lyndsey E. Collins-Praino

**Affiliations:** ^1^Cognition, Ageing and Neurodegenerative Disease Laboratory, School of Biomedicine, Faculty of Health and Medical Sciences, The University of Adelaide, Adelaide, SA, Australia; ^2^Medical Device Research Institute, College of Science and Engineering, Flinders University, Tonsley, SA, Australia; ^3^Allied Health and Human Performance, University of South Australia, Adelaide, SA, Australia; ^4^College of Medicine and Public Health, Flinders University, Bedford Park, SA, Australia

**Keywords:** cognitive training, Parkinson’s, serious games, co-design, dementia, brain training, cognitive impairment

## Abstract

Whilst Parkinson’s disease (PD) is typically thought of as a motor disease, a significant number of individuals also experience cognitive impairment (CI), ranging from mild-CI to dementia. One technique that may prove effective in delaying the onset of CI in PD is cognitive training (CT); however, evidence to date is variable. This may be due to the implementation of CT in this population, with the motor impairments of PD potentially hampering the ability to use standard equipment, such as pen-and-paper or a computer mouse. This may, in turn, promote negative attitudes toward the CT paradigm, which may correlate with poorer outcomes. Consequently, optimizing a system for the delivery of CT in the PD population may improve the accessibility of and engagement with the CT paradigm, subsequently leading to better outcomes. To achieve this, the NeuroOrb Gaming System was designed, coupling a novel accessible controller, specifically developed for use with people with motor impairments, with a “Serious Games” software suite, custom-designed to target the cognitive domains typically affected in PD. The aim of the current study was to evaluate the usability of the NeuroOrb through a reiterative co-design process, in order to optimize the system for future use in clinical trials of CT in individuals with PD. Individuals with PD (*n* = 13; mean age = 68.15 years; mean disease duration = 8 years) were recruited from the community and participated in three co-design loops. After implementation of key stakeholder feedback to make significant modifications to the system, system usability was improved and participant attitudes toward the NeuroOrb were very positive. Taken together, this provides rationale for moving forward with a future clinical trial investigating the utility of the NeuroOrb as a tool to deliver CT in PD.

## Introduction

Whilst Parkinson’s disease (PD) is primarily characterized as a motor disorder, many individuals also experience some degree of cognitive impairment (CI). Impairments in one or more cognitive domains may be observed even in early PD (<5 years) (Goldman et al., 2015), with many individuals also at risk of progression to mild CI (PD-MCI) and dementia (PD-D) (Litvan et al., 2012). Even in newly diagnosed individuals, PD-MCI is a common finding, with one study, the ICICLE-PD study, reporting that 42.5% of newly diagnosed individuals met criteria for PD-MCI using level 2 criteria (1.5 SDs lower than normative values) (Yarnall et al., 2014). By 3–5 years post-diagnosis, an estimated 20–57% of individuals qualify for diagnosis of PD-MCI (Caviness et al., 2007), with 20% converting to PD-D within 3 years (Saredakis et al., 2019). After 8 years, approximately 80% of individuals with PD develop PD-D (Aarsland et al., 2003). PD-D, classified as one of the Lewy Body dementias, is thought to be at least partially related to Lewy body pathology within limbic and neocortical areas (Smith et al., 2019), with Braak et al. (2005) reporting a correlation between declining scores on the Mini-Mental State Exam (MMSE) and higher neuropathologic state (Braak et al., 2005). Nevertheless, Lewy body pathology is only one contributor to the complex pathophysiology of PD-D, with other factors, including degeneration of neurotransmitter systems, the co-occurrence with Alzheimer’s disease-related pathology, and genetic factors also playing a role (for review, see Aarsland et al., 2021).

Cognitive impairment in PD may manifest in multiple domains, including executive function, attention, processing speed, visuospatial function, memory and verbal fluency, although not all domains are equally affected, particularly early in the course of the disease (Kehagia et al., 2010). Additionally, CIs, particularly those affecting executive function, are a key contributor to deficits in motor learning observed in PD, which may lead to increased gait and balance symptoms for individuals, and, ultimately, heightened risk of adverse events, such as falls (Olson et al., 2019). In support of this, the regulation of gait variability and rhythmicity, while an automatic process in healthy individuals, requires active attention in those with PD, with executive function deficits further exacerbating the increases in gait variability seen during dual-task performance (Yogev et al., 2005). Overall, CI represents the single biggest predictor of quality of life, mortality and caregiver burden for individuals with PD (Duncan et al., 2014).

Despite the prevalence and significant burden of CI in PD, however, interventions are limited. Whilst dopamine (DA) replacement therapies, such as levodopa, are effective at providing symptomatic relief for motor impairments, evidence for the treatment of CI is mixed, with some studies noting that intervention may paradoxically worsen cognition in certain domains (Schneider et al., 2013; Poston et al., 2016). The only currently approved treatment specifically for CI in PD is the use of cholinesterase inhibitors (Sun and Armstrong, 2021). These are, however, associated with prominent side effects (Ravina et al., 2005), variable efficacy between patients (Emre et al., 2014), and may even exacerbate the motor-symptoms of the disease (Collins et al., 2011). Furthermore, such therapies only address symptomatic presentation of established CI, unable to prevent or slow the development of cognitive dysfunction in PD (Cerasa and Quattrone, 2015). Consequently, current pharmacological interventions fall short in addressing CI in PD and, as such, interest has grown in non-pharmacological interventions, such as cognitive training (CT) (Guglietti et al., 2021; Sun and Armstrong, 2021).

Cognitive training is defined as training programs providing cognitive stimulation that offer structured practice on specific cognitive tasks (Clare and Woods, 2004). Multiple studies have established the efficacy of CT in improving or maintaining cognitive function in various neurological patient populations in areas such as global cognition, executive functions, learning, visuospatial abilities and memory (Sinforiani et al., 2004; Mohlman et al., 2011; Naismith et al., 2013; Petrelli et al., 2015; Folkerts et al., 2018). CT has also been shown to be effective both in cognitively healthy PD patients (Glizer and MacDonald, 2016), as well as those with PD-MCI (Reuter et al., 2012; Maggio et al., 2018), and the benefits may be maintained long-term up to 12 months (Petrelli et al., 2015; Bernini et al., 2019). Despite this, however, recent reviews of the literature as a whole have reported mixed results on the benefits of CT in PD (see, for example, Guglietti et al., 2021), with a Cochrane review evaluating the effectiveness of CT for PD-MCI and PD-D reporting no difference between CT intervention and control groups in measures of global or specific cognitive skills (Orgeta et al., 2020). It is important to note, however, that this review had strict inclusion criteria, capturing only randomized control trials.

The variability in outcomes for CT in the PD population may potentially be linked to differences in implementation strategies between programs, with many not optimized for use specifically in individuals with PD. In particular, the motor impairments observed in PD may represent a significant barrier to traditional CT modalities, with activities requiring a high level of manual dexterity, such as the use of pen and paper, which proves challenging for individuals with dyskinesia/akinesia (Thomas et al., 2017). Whilst the move from traditional pen-and-paper techniques to more computer-based programs may address some of these barriers, a 2010 survey found that nearly 80% of PC-users with PD have significant and severe difficulties using a computer due to their illness (Nes Begnum, 2010). In particular, muscle stiffness, inertia and tremor were frequent problems, resulting in significantly higher severe difficulties using a standard mouse (42%) and keyboard (27%) (Nes Begnum, 2010). Similarly, previous studies have shown one-third to half of computer users’ time is spent dragging a cursor via the mouse, which is considered a complex motor operation (Johnson et al., 1993) and represents a major obstacle to successful computer use for people with motor difficulties (Trewin and Pain, 1999; Kouroupetroglou, 2014). As such, this could represent a significant barrier to the delivery of CT in PD, potentially confounding the evaluation of outcomes. To address such barriers, assistive technologies and appropriate hardware, adapted for the PD population, are needed.

Engagement with the CT paradigm itself may also be key to the ultimate success of the intervention. In support of this, CT in a cohort of psychiatric patients determined engagement during training was a significant independent predictor of cognitive gains, irrespective of simple exposure (Harvey et al., 2020). Given that individuals with PD experience decreased reward sensitivity in an off-dopaminergic medication state, as well as increased apathy (Muhammed et al., 2016), this may be a particularly relevant concern for use of CT in this population. One strategy that may improve engagement is the addition of game-like features (gamification) into CT programs (Lumsden et al., 2016; Van De Weijer et al., 2019). This can be attributed to the incorporation of features such as high-score and reward incentives, narrative, personalization, self-directed challenge, exploration, free-play, competition and graphics into the training platform (Nagle et al., 2015). Gamification may also have benefits beyond improvement of engagement alone, with a systematic review of the literature of gamification in CT highlighting seven reasons researchers opted to gamify CT programs, including increased usability/intuitiveness for target age groups, increased ecological validity, increased suitability for the target disorder, and increased brain stimulation (Lumsden et al., 2016). The review concluded that gamified training is highly engaging and motivational and found evidence that gamification may be effective at enhancing CT in the elderly and ADHD populations (Lumsden et al., 2016). Similarly, recently, the Parkin’Play study showed enhanced global cognition scores after 24-weeks of individuals with PD participating in a home-based, gamified CT intervention (Van De Weijer et al., 2019).

To date, however, a system that targets the motor impairments that limit the use of traditional CT delivery methods in PD, while also incorporating elements of gamification that may improve engagement and treatment adherence, is lacking. In order to address this, we aimed to develop a novel “Serious Gaming” system, NeuroOrb, that would incorporate both assistive hardware and a custom gaming suite, designed to target the cognitive domains most affected in PD. Prior to deciding to embark on a large-scale trial to evaluate the potential cognitive benefits of CT delivered using the NeuroOrb system, we first engaged with individuals with PD in a reiterative co-design process, in order to evaluate the usability of the system in this population. Following incorporation of all feedback into system design, individuals were again asked to engage with the NeuroOrb system and evaluate the effectiveness of the changes. This manuscript details the outcomes of that co-design process, with future clinical trials planned to evaluate the cognitive benefit of the NeuroOrb system in individuals with PD.

## Participants and Methods

### Participants

Participants (*n* = *13*) were recruited from the community via Parkinson’s South Australia. Participation was voluntary and no incentive to participate was provided. Inclusion criteria included a prior diagnosis of PD by a registered neurologist and fluency in English. Exclusion criteria included significant hearing and visual impairments not corrected by glasses/contacts, a neurological disorder other than PD, or a previous diagnosis of a learning disability. All participants provided written informed consent prior to testing and the research conducted was approved by the Human Research Ethics Committee of the University of Adelaide (H-2020-214).

### The NeuroOrb System

**FIGURE 1:**
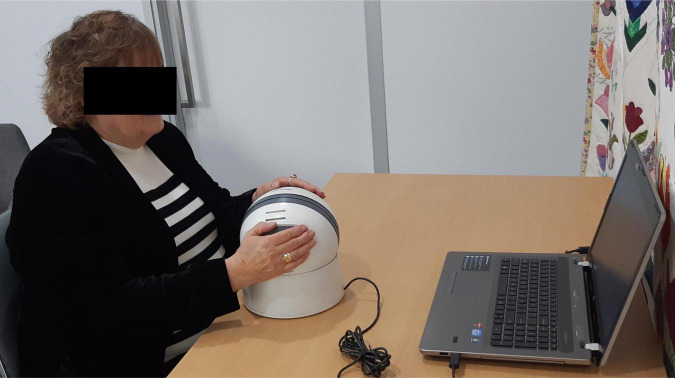
The Orby Controller, which consists of several features specifically adapted for motor dysfunction, including: (1) a spherical shape to allow for ergonomic bimanual control, (2) gray grip pads (partially obscured during use) and optional hand straps to support hand placement, (3) an adjustable sensitivity threshold, to prevent random movements from being interpreted as purposeful, (4) vibration to provide haptic feedback to the player, and (5) a large red selection button on the front of the controller to reduce the fine motor requirement (not shown).

The NeuroOrb serious gaming system involves two main components. The first is the use of an assistive hardware via the ‘Orby’ controller (Figure 1). Orby is an innovative novel controller that was custom co-designed by one of the authors of the current study (DH) to address barriers associated with motor dysfunction, such as reduced fine motor control and tremor (Walker and Hobbs, 2014; Hobbs et al., 2019). The spherical “Orb” part of the controller is 200 mm in diameter and the top of the controller is 230 mm above the table surface. The spherical shape allows for ergonomic bimanual control, with grip pads on either side to indicate ideal hand placement. Importantly, the sensitivity of responsiveness can be adjusted for the individual, with the movement recognized as “purposeful” altered to take into account the extent of motor impairment. This is particularly important for PD patients with resting tremor or drug-induced dyskinesias, as unintentional tremors can be ignored, leaving only intentional movements to direct the controller during CT. The controller also includes vibration, which allows for haptic feedback triggered by actions within each game. Finally, the large red button for selection minimizes the requirement for fine motor control that may be required with devices such as iPad or keyboard keys, which may otherwise be a concern for PD patients. Orby has previously been trialed successfully in individuals with hand impairments due to disability, such as cerebral palsy and in adults post-stroke (Hobbs et al., 2017), but has not previously been trialed in individuals with PD.

**FIGURE 2:**
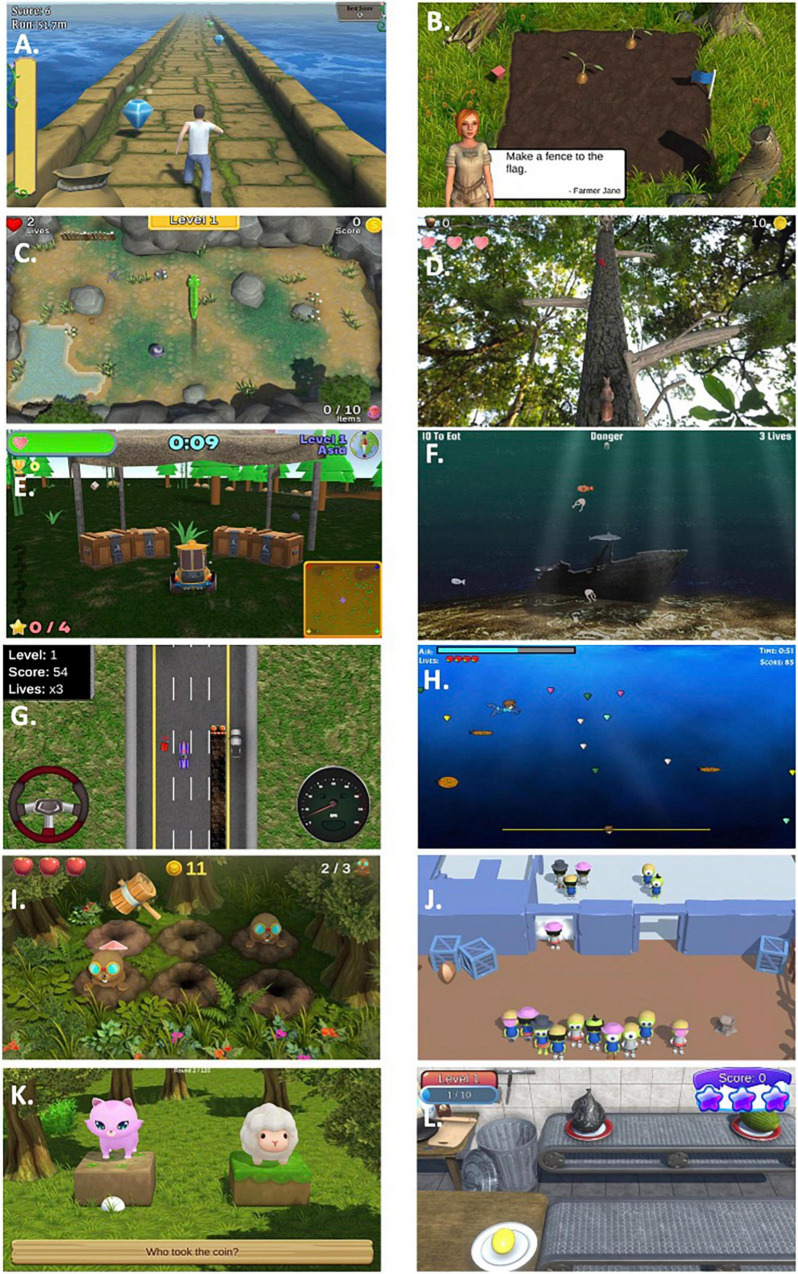
Representative screenshots from each of the games included within the NeuroOrb suite: **(A)** A Bridge Too Far; **(B)** Farm Quest; **(C)** Snake; **(D)** Squirrel; **(E)** Sunday Driver; **(F)** Marine Life; **(G)** Driving Maniac; **(H)** Swimma; **(I)** Whack-a-Mole; **(J)** Munchkinis; **(K)** Who’s the Boss?; **(L)** Chow Time!

Secondly, to improve user engagement, a custom serious gaming suite was designed to target the cognitive domains most affected in PD, including executive function (working memory, attention, cognitive flexibility, problem solving), visuospatial function and learning (Watson and Leverenz, 2010; Figure 2). Some of these games were adapted from existing games designed by one of the authors of this study (DH). Other games were developed specifically for the NeuroOrb system. Each game is described below, with further information on the cognitive domains that each game targets summarized in Table 1. Due to the nature of gameplay, several of the games encompass training in multiple domains. This gamification of the CT paradigm introduces elements of high-user control, self-directed challenge, exploration and free-play, which have previously been shown to improve outcomes in home-based CT compared to more automated task delivery (Nagle et al., 2015).

**TABLE 1:** Overview of primary and supplementary cognitive domains trained in the gaming suite and game descriptions.

Game	Primary rationale	Supp 1	Supp 2	Supp 3	Game description
A Bridge Too Far	Working Memory	Cognitive Flexibility/Set Shifting	Visuospatial		A running game that requires the player to collect coins, cans of soda (for energy) and the correct color gem, while navigating a never-ending path and avoiding gaps.
Farm Quest	Problem Solving/Abstract Reasoning	Planning	Attention		A puzzle game that requires problem solving skills to separate different farm produce into like groupings and reach a flag on the other side of a vegetable patch.
Snake	Visuospatial Function	Avoidance Learning	Attention		A snake character needs to be navigated within an arena, avoiding obstacles, boundaries and its own body, whilst collecting different “orbs.” Additionally, an eagle intermittently flies across the arena requiring the player to decide if they will risk their life to collect the orbs or seek safety in a nearby lake.
Squirrel	Attention	Working Memory	Cognitive Flexibility/Set Shifting	Visuospatial	A squirrel character requires navigation around a never-ending tree, avoiding branches, with speed and number of obstacles increasing as the player progresses. Prior to the start of each level, players are presented with a “shopping list” of colored berries and the number of each that they are required to collect. Players must avoid all other colored berries not specified for collection.
Sunday Driver	Attention	Working Memory	Spatial Navigation	Cognitive Flexibility	A driving game that requires players to navigate a large area to find and collect different characters that are spread around the course through trial and error, and to return them to a central tent.
Marine Life	Attention/Working Memory	Cognitive Flexibility/Set Shifting	Avoidance Learning	Spatial Navigation	A deep ocean game that requires different sea creature characters to be navigated around to eat a specified number of other sea creatures before progressing to the next level. Each level includes a “danger” creature (or creatures) that the player must avoid, with the player’s character and those they are asked to eat and avoid changing with each level.
Driving Maniac	Visuospatial	Attention			A vertical-scrolling driving game that involves navigation of a car along a road where the player must avoid obstacles such as other cars, oil slicks and roadblocks, whilst collecting extra lives and fuel-tanks. Speed increases with distance traveled, to increase the difficulty.
Swimma	Attention	Working Memory	Problem Solving/Abstract Reasoning	Cognitive Flexibility/Set Shifting	A side-scrolling game that requires navigation of a scuba diver character through a constantly moving underwater environment, whilst avoiding other sea creatures, collecting certain gems, avoiding a particular gem, and collecting air bubbles and extra lives to stay alive.
Whack-A-Mole	Attention	Working Memory	Sequence Learning		A game that requires players to hit dirt burrows with a hammer in the same sequence that moles appear and then hide, to reveal each mole. The number of burrows increases with each stage, the number of moles appearing in a sequence increases, and the rate of appearance varies between moles (e.g., first and second moles might pop up quickly, but the third may be slightly delayed).
Who’s the Boss?	Problem Solving/Abstract Reasoning	Working Memory	Risk-Taking Behavior		A puzzle game that presents the player with a pair of characters (e.g., Cat/Sheep) and asks them to guess who they believe the “boss” is, with feedback (correct/incorrect) provided instantaneously. Subsequent pairs of characters (e.g., Penguin/Chicken) are then presented, and through a series of exposures of different combinations of paired characters, the player must determine the correct hierarchy of characters, receiving coins depending on the number of paired exposures they required to make their guess. The player can also choose to bet on their confidence in their final decision.
Munchkinis	Abstract Reasoning	Cognitive Flexibility/Set Shifting			A puzzle game requiring the guidance of “Munchkinis” characters through a series of gates, in order to guide them home. Each level involves a series of gates (2 or 4) that allow entry based on a particular trait (e.g., glasses vs. no glasses, hat vs. hair, etc.). The player must use trial and error to determine the sorting criteria and allocate each Munchkinis through their respective gate.
Chow Time	Response Inhibition	Attention	Working Memory		Players are presented with a moving conveyer belt with various foods/items and must sort the edible food (e.g., melon) from the non-edible items (e.g., an old boot). To select, players must make an intentional selection of the edible food by moving the controller toward the second conveyer belt to collect; however, no movement is required when faced with inedible food. The conveyer belt increases in speed with each level.

### Assessment of NeuroOrb

In order to assess overall acceptability of NeuroOrb, the well-validated System Usability Scale (SUS) was administered both pre- and post-modification (Peres et al., 2013). Additionally, to investigate participants’ perceptions of the Orby controller, games catalog and NeuroOrb system overall, two surveys were developed in-house. These surveys were specifically developed for the purposes of this co-design trial and have not been externally validated.

The first of these assessed feedback on the NeuroOrb system, with questions regarding whether individuals enjoyed the system as a whole (calculated as % responding yes), whether they found the system challenging to use (calculated as % responding yes), how enjoyable/difficult they found the games/Orby controller to use (rated on a scale of 1–10, with 1 = very poor/easy and 10 = very challenging/enjoyable), and their confidence in whether they thought that routine engagement with the NeuroOrb system would result in improvement or maintenance of cognitive function (rated on a scale of 1–10, with 1 = not at all confident and 10 = very confident). This survey was repeated both pre- and post-modification of the NeuroOrb system, with an additional question added post-modification asking whether participants felt that their comments had been addressed.

The second survey, given both pre- and post-modification of the NeuroOrb system, focused on the content and usability of the individual games themselves. Participants were asked whether they enjoyed each game, whether they found the game challenging, whether they found the instructions for each game clear, and whether they found the game easy to play (all calculated as % responding yes). Further, for each game, participants were asked to rate their enjoyment of the game, as well as how difficult they found each game (each rated on a scale of 1–10, with 1 = very poor/easy and 10 = very challenging/enjoyable). Finally, features of each game (i.e., color/animation/sound) and features of the game control were both rated on a scale of 1–3, with 1 = poor/not good, 2 = average and 3 = good.

### Procedure

**FIGURE 3:**
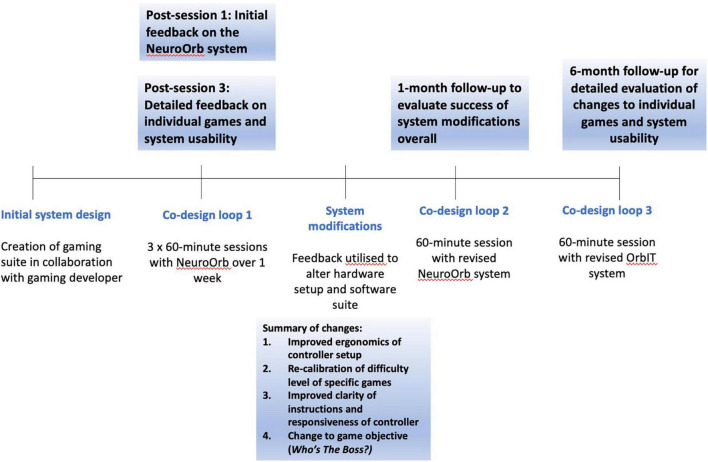
A timeline of the co-design trial. The NeuroOrb system was first developed in collaboration with a gaming developer (BW). Participants were then invited to participate in three 60-min sessions over the course of a week, spaced 48 h apart. Extensive survey and verbal feedback was collected at the end of the first and third sessions. This feedback was then used to make significant changes to both the hardware and software of the NeuroOrb system. Following these changes (∼1-month post-exposure), participants were invited back for another 60-min gaming session and were encouraged to provide initial feedback on the changes made. They were then invited back a second time (∼6 months post-exposure) for a further 60-min session with the NeuroOrb system, followed by the completion of detailed feedback about the gaming suite and system as a whole.

Eligible participants (*n* = *13*) attended the Brain and Body Fitness Studio (BBFS) at Parkinson’s South Australia and completed three 60-min sessions with NeuroOrb over the course of a week (Figure 3). All participants were tested in the “on”-stage of their medications (i.e., the period in which motor symptoms are well-controlled by the medication). While caregivers were not present during the training sessions, participants were supervised during game play by a member of the research team with prior expertise in using the NeuroOrb system. A pre-exposure battery, consisting of demographic questions, the Mini-Mental State Examination (MMSE) to assess baseline cognitive function (Folstein et al., 1975), the Parkinson’s Disease Questionnaire-39 (PDQ-39) to assess disease-specific quality of life (QoL; Jenkinson et al., 1997) and the Geriatric Depression Scale (GDS) as a self-report measure to assess depression in older adults (Pocklington et al., 2016), was administered on day 1, followed by a 60-min NeuroOrb session. Day 2 involved 60-min of supervised gameplay and day 3 involved 60-min of gameplay, followed by a post-exposure battery. This post-exposure battery consisted of a series of questionnaires on previous game experience, system feedback and individual game feedback. Additionally, the System Usability Scale (SUS) was administered to assess overall acceptability of NeuroOrb (Peres et al., 2013).

Participants were provided with a brief controller demonstration and written instructions for each game and assistance was provided if requested during the first session to help familiarize individuals with the controller and game objectives. Play for each game was restricted to 15 min over the course of the week to ensure an even spread of training across multiple domains. Feedback was collected via both written surveys and verbal communication across the three sessions and adjustments to the gaming suite and controller setup were made based on this feedback.

Participants were invited back 1-month and 6-months later for two additional 60-min sessions with the adjusted setup to provide feedback on implementation of suggested changes. The 1-month session was conducted to allow individuals to assess the success of changes overall, while the original gameplay experience was still fresh in their minds. Conversely, the 6-month session was conducted to allow enough time to have passed from the initial system engagement to minimize carry-over effects that could potentially bias the final system and games evaluation. During co-design loop 2 (∼1-month post-engagement), follow-up feedback on the overall changes to NeuroOrb, as well as assessment of overall system enjoyment, was collected. During co-design loop 3 (∼6 months post-engagement), individuals again completed the SUS and were asked for feedback on the individual games. Throughout all sessions, observations were recorded and verbal feedback was noted by trained members of the research team.

### Statistical Analysis

Demographic data are presented as either Mean (±SD) or Range/Proportion, depending on the variable. For rating scales, each item was scored on either a 10-point or 3-point Likert scale. Data are presented as Mean (±SD), with the exception of measures presented as the percentage of participants endorsing “yes” for a particular measure. For pre- to post-modification improvement, the absolute increase in percentage for percentage-based measures and the absolute increase in points for scale-based measures was calculated. Data were analyzed using SPSS (Version 26).

## Results

### Participant Demographics

Thirteen participants were included in the pilot (6M/7F, Mean Age 68.15 ± 8.54) years), with an average disease duration of 8 ± 5.43 years. Demographics are summarized in Table 2. Twelve of the 13 participants completed all 3 sessions of the CT training period, with 1 completing 2/3 due to time constraints. Eleven of the 12 participants (92%) attended the 1-month follow-up and nine of the 12 participants (75%) attended the 6-month follow-up. Participants did not appear to be cognitively impaired, with MMSE scores all > 27 (Mean 29 ± 0.82). Despite not scoring in the CI range on the MMSE, a subjective survey revealed participants commonly reported cognitive concerns, including remembering events (30.1%), remembering information (38.5%), paying attention (15.4%), learning new tasks (15.4%), remembering words (15.4%) and managing day-to-day tasks (15.4%). In addition, 38.5% self-reported experiencing motor difficulties.

**TABLE 2:** Demographic and patient data (*n* = *13*), Mean ± SD.

Patient Data	Mean ± SD	Range/Proportion
Age (Years)	68.15 ± 8.54	48–81
Gender (M/F)	–	6/7
Years Since Diagnosis	8 ± 5.43	1–19
MMSE	29 ± 0.82	–
GDS	3.42 ± 4.08	–
PDQ-39 Summary Index	21 ± 11.25	–
Affected Side (Right/Left/Equal)	–	2/6/5
Dominant Hand Affected	–	7/13
DBS Surgery	–	2/13
Time Since Medication (hours)	1.85 ± 1.11	–

Unfortunately, neither the MDS-Unified Parkinson’s Disease Rating Scale (MDS-UPDRS) nor Hoehn and Yahr (HY) staging was available for the current sample. However, previous work has suggested that the median disease duration for HY staging is 4 years for Stage 1, 5 years for Stage 2, 7 years for Stage 3, 10 years for Stage 4 and 14 years for Stage 5, with individuals with disease duration from 6 to 10 years, which represents the majority of individuals in the current sample, evenly represented across all five stages (Skorvanek et al., 2017). From observations carried out during game play with the NeuroOrb system, motor impairments did not appear to prevent any participant from actively engaging with the system, and no participant expressed concerns to this effect in their comments.

At baseline, PD participants did not report a decrease in health-related QoL in any domain measured by the PDQ-39 compared with normative data (Jenkinson et al., 1997). Furthermore, participants mean GDS (3.42 ± 4.08) did not indicate depression, with scores > 11 indicating depression on this measure (Mondolo et al., 2006).

With relation to previous game experience, 92.3% of participants reported playing games, with 53.8% reporting a frequency of 1 or more times daily. The preferred gaming formats reported were card games (76.9%), word and number games (76.9%) and puzzle/tile/board games (38.5%). In terms of computerized games, 30.8% reported playing games in an online format, whilst only 1 participant (7.7%) reported a preference for video games, indicating the sample group had minimal experience with computerized video games prior to engaging in the co-design process.

### System Feedback- Co-design Loop 1

#### Session 1: Initial Feedback

After one session, 91% of participants reported that they enjoyed the NeuroOrb gaming system, with an average rating of 7.58 ± 2.19/10 (Table 3). 100% of participants reported finding the games challenging, with the average difficulty level of the games rated as 6.25 ± 1.29/10. However, the games were still reported as being enjoyable, with an average overall enjoyment rating of 7.75 ± 2.18/10. Furthermore, participants reported a high degree of confidence in the ability of the NeuroOrb system to improve/maintain cognitive function (7.92 ± 1.78/10). One participant reported that they would not use the system at home, one reported occasional use, six reported use several times a week and five reported the likelihood of daily use.

**TABLE 3:** Session 1 – Initial feedback on the NeuroOrb system.

Initial Feedback		Mean ± SD
Initial Enjoyment	% Yes	91.7%
	Games	7.75 ± 2.18
	NeuroOrb System	7.58 ± 2.19
Challenging	% Yes	100%
Difficulty	Games	6.25 ± 1.29
	Controller	5.83 ± 1.85
Confidence	Likelihood of improvement/maintenance of cognitive function	7.92 ± 1.78

*%, percent of participants who answered “yes.” Scores are based on a scale of 1–10 (1 = very poor/easy, 10 = very challenging/enjoyable). n = 12, Mean ± SD.*

**FIGURE 4:**
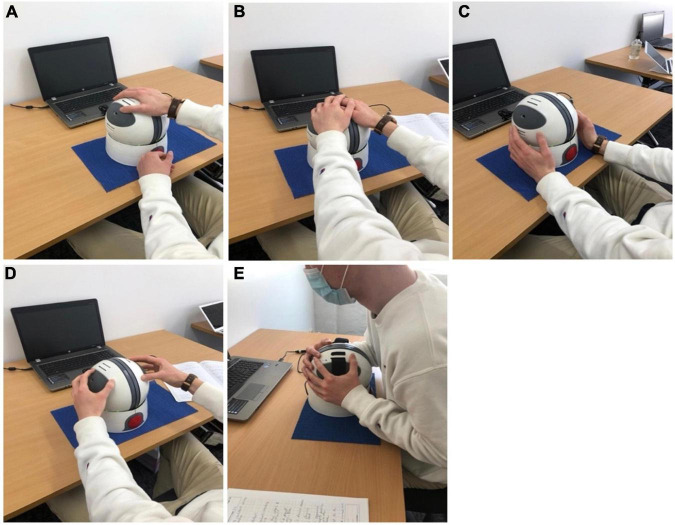
Despite being shown a traditional grip (one hand over each grip pad) in the initial session, following additional exposures to the Orby controller, participants adopted several different techniques to control the device, including one handed **(A)**, upper hold **(B)** or lower hold **(C)**, fingertips **(D)** and bear grip **(E)** for those with more prominent motor dysfunction.

With regards to accessibility of the Orby controller itself, this received only a moderate rating (5.83 ± 1.85/10) after the first exposure to the system. Observationally, participants appeared to become more comfortable with controller use by the second exposure and proficient by the third session, with one participant noting, “*the gaming platform was not too awkward to operate once I became used to the tension of the [NeuroOrb] i.e., not to be too forceful gripping*.” Encouragingly, with repeated use, participants also optimized their own use of the Orby controller. Despite being shown a traditional grip (one hand over each grip pad) in the initial session, throughout the trial and across different games, participants adopted several different techniques to control the device, including one handed (Figure 4A), upper hold (Figure 4B) or lower hold (Figure 4C), fingertips (Figure 4D), and bear grip (Figure 4E) for those with more prominent motor dysfunction (Figure 4).

#### Individual Game Feedback: Pre-modification

Following completion of the third session of play, feedback was collected on the individual games. Data were separated into two categories: content (enjoyment, interest, challenge, difficulty and features) and usability (instructions, ease of play and controls). An overall system rating was also obtained. For each game, the number of participant responses received depended on whether the game was played during their sessions. Overall ratings for each game are summarized in Table 4.

**TABLE 4:** Individual game feedback, pre-modification.

	Content						Usability			Overall rating
	Res.	Enjoyed (% yes)	Enjoyment rating (Av interest + Enjoyment)/10	Challenge (% yes)	Difficulty rating/10)	Features (color/animation/sound)/3	Clear instruction (% yes)	Ease of play (% yes)	Controls/3	
A Bridge Too Far	13	100%	7.42 ± 1.82	84.6%	7 ± 0.0	2.81 ± 0.27	69.2%	100%	2.62 ± 0.51	**7.69 ±1.60**
Farm Quest	11	36.4%	5.73 ± 2.31	100%	7.36 ± 1.63	2.77 ± 0.44	70%	54.6%	2.7 ± 0.48	**5.5 ±2.36**
Squirrel	12	91.7%	7.17 ± 1.36	81.8%	6.33 ± 1.72	2.73 ± 0.46	100%	81.8%	2.64 ± 0.67	**7.17 ±1.34**
Snake	12	41.7%	4.54 ± 1.83	75%	6.67 ± 2.23	2.64 ± 0.55	70%	33.3%	1.82 ± 0.75	**4.83 ±1.80**
Sunday Driver	9	66.67%	6.44 ± 1.97	100%	7.44 ± 1.24	2.59 ± 0.52	22.2%	22.2%	2.62 ± 0.52	**6.33 ±2.24**
Marine Life	11	90.9%	7 ± 2.23	90%	6.73 ± 1.95	2.83 ± 0.37	88.9%	72.7%	2.5 ± 0.53	**7.27 ±2.15**
Swimma	7	85.7%	6.71 ± 1.50	85.7%	6 ± 1.83	2.56 ± 0.54	66.7%	57.1%	2.33 ± 0.52	**6.43 ±2.37**
Driving Maniac	12	100%	7.71 ± 2.18	100%	7.33 ± 1.23	2.52 ± 0.52	85.7%	75%	2.46 ± 0.53	**7.83 ±1.80**
Whack-A-Mole	10	80%	7.55 ± 1.58	80%	7.2 ± 1.40	2.73 ± 0.45	100%	88.9%	2.33 ± 0.82	**7.7 ±1.77**
Munchkinis	7	85.7%	7.64 ± 2.33	100%	8 ± 1.41	3 ± 0.0	100%	57.1%	3 ± 0.0	**7.57 ±1.90**
Who’s the Boss?	10	70%	5.85 ± 2.16	100%	7.3 ± 1.49	2.67 ± 0.49	66.7%	80%	2.67 ± 0.5	**6.3 ±1.34**
Chow Time	10	80%	6.3 ± 2.64	30%	4.2 ± 1.75	2.63 ± 0.51	100%	100%	2.75 ± 0.46	**6.4 ±2.84**

*Res, respondents, % indicates percent of participants who answered “yes.” Ratings are on a scale of 1–10 (1 = very poor/easy – 10 = very challenging/enjoyable). Features and controls are on a scale of 1–3 (1 = Not Good, 2 = Average, 3 = Good). For all ratings, the table presents Mean (±SD).*

Overall, the response to the games was positive, with all games (except Snake) receiving an above average (>5) overall rating. These results were probed further based on feedback regarding content and usability. Content was broken down into enjoyment, difficulty and game features. In terms of enjoyment, the majority of games were considered enjoyable, with >90% of participants reporting enjoyment of *A Bridge Too Far* (7.42 ± 1.82/10), *Squirrel* (7.17 ± 1.36/10), *Marine Life* (7 ± 2.23/10) and *Driving Maniac* (7.71 ± 2.18/10) and >50% of participants reporting enjoyment of *Swimma* (6.71 ± 1.50/10), *Sunday Driver* (6.44 ± 1.97/10), *Whack-A-Mole* (7.55 ± 1.58/10), *Munchkinis* (7.64 ± 2.33/10), *Who’s the Boss?* (5.85 ± 2.16/10) and *Chow Time*! (6.3 ± 2.64/10). Games with a below average (<50%) number of participants reporting enjoyment included *Farm Quest* (5.73 ± 2.31/10) and *Snake* (4.54 ± 1.83/10).

With regards to difficulty, we sought to balance achieving an effective challenge with reducing the “cognitive cost” of games by ensuring that all games fell into a difficulty level of between 6 and 8 on a 10-point scale. All games were within this range, with the exception of *Chow Time!*, with only 30% of participants reporting this game to be challenging. This was corroborated with observations and verbal feedback, with participants reporting the speed of the conveyer belt started off “*too slow”* and did not become challenging until at least level 5. Conversely, although still within an acceptable range, *Munchkinis* was considered the most difficult game included in the suite. Based on observations during the first three sessions, this also appeared to be related to progression. For example, the first level of *Munchkinis* involved the sorting of features based on 2 criteria, before level 2 progressed to sorting based on multiple characteristics. This progression was reported to be “*too steep*,” with only one participant observed to successfully complete the second stage.

In terms of game features, including the use of color, animations and sound, all games were rated > 2.5 on a three-point scale, with no notable comments or observations made with regards to the visual features of the games’ design. Interestingly, one participant did comment on the reliance on color for sorting in many of the games, particularly *Squirrel*, as this would be a potential barrier for implementation in those with color blindness. Whilst this feedback was not directly addressed in the initial round of changes, future adaptations could include the use of shape, rather than color, to overcome this particular concern.

Usability of the games was assessed based on the ease of play, clarity of instructions provided and controller responsiveness for each game. *A Bridge Too Far* and *Chow Time!* were considered easy to play by 100% of participants. Whilst *Chow Time*’s ease is likely attributed to the slow progression of the game, *A Bridge Too Far* appeared to be quite challenging for participants based on observations. This may be because the game is the first listed in the suite and, as such, all participants chose to begin with this game. This meant that, when observers were guiding participants through the features and use of the controller for the first time, it was via this game. This may have led users to feel particularly supported in how to play the game, raising their confidence level and inflating their perception of the ease of the game. Conversely, games which scored poorly (<60%) for ease of play included *Farm Quest, Snake, Sunday Driver, Swimma* and *Munchkinis*. Based on participant comments, this was attributable to poor clarity of the instructions and poor responsiveness of the controls. For example, participants specifically commented “*instructions not clear*” or “*not easy to follow*” for *Farm Quest*. For *Sunday Driver*, participants commented it was “*not clear had to go to center tent first*.” Whereas comments for *Snake* included “*didn’t seem to respond to controls”* and “*controlling the snake was difficult, needs refining*.”

Concerningly, upon initial rating, only half of all games received an endorsement of >85% for clarity of instructions. For five of the remaining games (*A Bridge Too Far, Farm Quest, Snake, Swimma* and *Who’s the Boss?*), a more moderate percentage of participants (65–70%) reported that the instructions were clear. Overall, participants reported that “*having instructions built into the program would be ideal*.” This discrepancy was also noticed in observations, with considerable guidance required initially to assist participants in identifying the objectives and features of the games.

#### Overall Usability: Pre-modification

Following the third session, participants were asked to complete the SUS to assess overall usability of the NeuroOrb System. Results are summarized in Table 5. Interpretation of usability based on standard SUS guidelines resulted in an overall score of 65.58/100. According to the SUS grading system, this is considered below the average score of 68 (Gomes and Ratwani, 2019). Furthermore, using the scale developed by Sauro (2011), this score equates to a grade of “C” (approximately 42nd percentile) (Sauro, 2011).

**TABLE 5:** Results of the system usability scale questionnaire: pre- versus post-modifications.

Scores are based on a scale of 1–5 (1 = Strongly Disagree, 5 = Strongly Agree). Mean ± SD		
	Pre-modification (*n* = 13)	Post-modification (*n* = 9)
	
System Usability Scale – Questions	Mean ± SD	Mean ± SD
I think that I would like to use the NeuroOrb System frequently	3.23 ± 0.6	3.67 ± 0.71
I found the NeuroOrb System unnecessarily complex	1.92 ± 0.86	1.67 ± 0.71
I thought the NeuroOrb System was easy to use	3.46 ± 0.78	4.00 ± 1.12
I think that I would need the support of a technical person to be able to use the NeuroOrb System	2.08 ± 0.95	2.11 ± 1.05
I found the various functions of the NeuroOrb System were well integrated	3.46 ± 0.88	3.78 ± 0.44
I thought there was too much inconsistency in the NeuroOrb System	2.39 ± 1.04	1.56 ± 0.53
I would image that most people would learn to use the NeuroOrb System very quickly	3.77 ± 0.93	4.00 ± 1.00
I found the NeuroOrb System very cumbersome to use	2.38 ± 0.96	2.11 ± 1.09
I felt very confident using the NeuroOrb System	3.39 ± 0.96	4.00 ± 1.00
I needed to learn a lot of things before I could get going with the NeuroOrb System	2.31 ± 0.95	2.11 ± 1.20
**Overall score**	**65.58/100**	**74.17/100**

Based on participant feedback, this may, at least in part, be due to general setup/handling issues, with several participants noting comments such as, “*I think the device needs to be anchored to a base, so it doesn’t move about the table*.” Fatigue/discomfort may also have played a role, with two participants commenting that their shoulders/arms became tired or sore during the session. Additionally, one participant ended 2/3 of their sessions 5 min early due to fatigue.

### Modifications Made Following Co-design Loop 1 Feedback

To address the issues identified during the co-design, significant alterations were made, including: (1) improved ergonomics of the controller setup, (2) re-calibration of the difficulty level of tasks within specific games, (3) improved clarity of instructions and responsiveness of controller and, in the case of “*Who’s the Boss?*” specifically, (4) a change to the game objective.

#### Changes to Ergonomics of Controller Setup and Feedback

The controller setup itself was altered, in order to improve user experience and decrease fatigue associated with extended use. This included the introduction of a grip mat, improving how the controller remained in place on the table, as well as the introduction of the optional straps that the controller was designed with, in which participants could place their hands for improved grip/handling of the controller. The straps also enabled participants to “rest” their hands to reduce fatigue and shoulder strain whilst still maintaining control. This reduction of shoulder/arm fatigue was further enhanced through the use of height-adjustable ergonomic chairs with arm rests to provide additional support. Overall, changes to the ergonomics of the set-up and controller were well received, as demonstrated by comments such as, “*mat beneath controller helps*;” “*straps help when moving object on game i.e., are great help when having to just select*” and “*using straps and rubber mat was good*.” Most encouragingly, one participant noted, “*the controller was much easier than a mouse and keyboard*” and mentioned that, although they hadn’t taken their medication yet, “*it was still easy to navigate on the [NeuroOrb].”* This was also reflected in the follow-up survey where, on a scale of 1 (much worse) to 10 (much better), changes to the controller itself were rated 7.64 ± 1.75/10.

#### Changes to the Difficulty Level of Games and Feedback

In order to ensure that all games fell in our ideal range of 6–8/10, modulation of task difficulty was made for both *Chow-Time!* and *Munchkinis*. For *Chow-Time!*, the initial speed of the conveyer belt, and accordingly the required processing speed, was increased. Following modifications, the percentage of participants that reported finding the game challenging increased 37% (from 30 to 67%) and the difficulty rating increased 2.8 points (from 4.2 ± 1.75/10 to 7.0 ± 1.00/10), bringing the game in line with our target difficulty. This was well received, as reflected in verbal feedback in the follow-up session, with participants commenting “*starting speed is better,”* which “*made it more interesting*.” Additionally, the overall rating of the game increased 2.93 points (from 6.4 ± 2.84/10 to 9.33 ± 0.58/10). For *Munchkinis*, conversely, an additional level comparable to the first one was added, in order to allow the participants more time to identify and familiarize themselves with the sorting criteria. Following these changes, observers noted an increase in the number of participants reaching the final level at follow-up. This was accompanied by a 1.33 point decrease in difficulty rating (from 8.0 ± 1.41/10 to 6.67 ± 2.31/10), as well as a 1.76 point increase in overall rating (from 7.57 ± 1.90/10 to 9.33 ± 0.58/10). Interestingly, at post-modification rating, three games fell just below the target range of 6–8: *A Bridge Too Far* (5.22 ± 1.56/10), *Snake* (5.75 ± 1.71/10) and *Driving Maniac* (5.75 ± 3.40/10). It is important to note, however, that this was based on a small number of ratings and may be influenced by previous exposure effects.

#### Improved Clarity of Instructions/Responsiveness of Controller and Feedback

**FIGURE 5:**
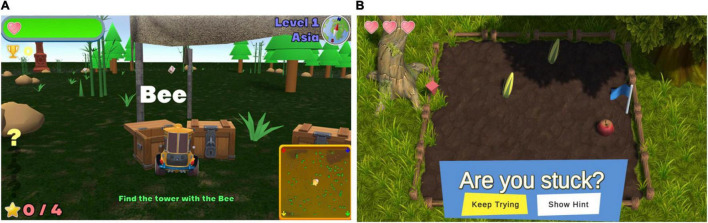
**(A)** One of the changes made to Sunday Driver included the addition of prompts to instruct individuals based on their gameplay. For example, players would be reminded of the goal if they remained stationary for too long. **(B)** One of the changes made to Farm Quest included the addition of a time-activated hint trigger, offering the player the option of a hint for the next move.

One of the main changes implemented across ***all*** games was the incorporation of instructions into each game’s menu, rather than in a separate written booklet. Additionally, images were included in the instructions to assist in familiarizing the participants with elements they encounter during game play. This appeared to have an immediately beneficial effect, with participants commenting that they had not previously recognized elements of gameplay prior to reading the new on-screen instructions. The addition of explanatory pictures seemed to be a key driver of this, with one participant noting, “*I love the pictures in the instructions so I knew what to look for*.” Of all games, *Sunday Driver* received the poorest rating of instruction clarity at baseline (22.2%), a finding corroborated by observers, who needed to provide considerable guidance for players. Accordingly, for this game, instructions were incorporated to appear during game play itself, rather than solely all at the beginning. This allowed the player to be guided by instructions based on their game play (e.g., if they stayed stationary, a prompt would appear instructing them of their next goal) (Figure 5A), leading to enhanced clarity about what was required at each stage of game play and better engagement with the game during a follow-up session. This was reflected in the post-modification ratings, with an absolute increase of 45% (from 22 to 67%) in the percentage of individuals reporting that the instructions were clear and an absolute increase of 78% (from 22 to 100%) in the percentage of individuals endorsing the game as easy to play.

Overall, changes to the instructions were well-received at follow-up sessions, with one-third of games (*A Bridge Too Far, Snake, Sunday Driver* and *Who’s the Boss?)* showing an improvement in the percentage of individuals reporting that the instructions were clear compared to baseline. A further one-third of games (*Squirrel, Swimma, Munchkinis*, and *ChowTime!)* did not show a change from baseline, but this is likely reflective of the fact that all of these, with the exception of *Swimma*, had already obtained a 100% rating for instruction clarity at baseline. For the remaining one-third of games (*Farm Quest, Marine Life, Driving Maniac*, and *Whack-a-Mole)* that showed a decrease in the percentage of individuals reporting that instructions were clear, this may be biased by the small number of ratings (just four per game).

Given that three of these four games, with the exception of *Marine Life*, showed an improvement in overall rating compared to baseline, however, it may also indicate that factors other than instruction clarity were driving game feedback. In line with this, for *Farm Quest*, participants attributed their difficulty in understanding what was required to progress between levels not to the instructions, but instead to their perceived ability, with one stating, “*problem was mainly me I feel*” and going on to state that their dissatisfaction with the game was “*just due to my comprehending or not understanding the rules*.” Given that *Farm Quest* involves problem solving and abstract reasoning, this may reflect particular challenges with this cognitive domain in individuals with PD (Beatty and Monson, 1990; Cronin-Golomb et al., 1994; Young et al., 2010). As such, in order to minimize frustrations, a time-activated hint trigger was added, offering the player the option of a hint for the next move if required (Figure 5B). Encouragingly, this was associated with an absolute increase of 64% (from 36 to 100%) in the percentage of individuals reporting that they enjoyed the game and an increase of 2.25 points in the overall rating of the game (from 5.5 ± 2.36/10 to 7.75 ± 0.96/10).

Modification was also made to the controller sensitivity and responsiveness. This modification was particularly noteworthy for *Snake*, which was the only game to initially receive a below average (<2) rating (1.82 ± 0.75/3) of the usability of specific controls within the game. This translated to poor measures of enjoyment, with only 41.7% of participants reporting that the game was enjoyable and an overall rating of just 4.54 ± 1.83/10, the lowest for any game. Participants were not able to pass the first level and did not attempt to play the game after the first exposure. Accordingly, several changes were made, including optimization of the controller sensitivity to movement and directional changes, a reduction in speed of the snake, an increase in arena size and the removal of obstacles from the first three levels, in order to give the player a larger margin of error to make directional decisions and more time to adjust to the controls. These changes were well received, with participants reporting they could “*feel [the controller] responding better,”* and the rating of the usability of controls within the game increasing 0.93 points (from 1.82 ± 0.75/3 to 2.75 ± 0.50/3). Ratings of the game itself also changed strikingly post-modification, with an absolute increase of 58% (from 42 to 100%) in the percentage of individuals endorsing the game as enjoyable and an absolute increase of 67% (from 33 to 100%) in the percentage of those reporting that the game was easy to play. Similarly, the enjoyment rating of the game increased 2.96 points (from 4.54 ± 1.83/10 to 7.5 ± 0.58/10), with overall rating increasing 2.42 points (from 4.83 ± 1.80/10 to 7.25 ± 0.96/10).

#### Change to Game Objective and Feedback

*Who’s the Boss?* was originally the least successful of all games in the catalog, receiving an average enjoyment score of just 5.85 ± 2.16/10 and a difficulty rating of 7.3 ± 1.49/10. It also received the most negative comments of all games, with participants reporting the game to be “*frustrating*,” “*hard to find a pattern*” and “*just a bit of random guesswork most of the time*.” Accordingly, the game was re-pitched entirely, from a confusing and monotonous task based on reinforcement learning principles to a hierarchical structure that required problem solving/abstract reasoning for individuals to identify “the boss” based on the lowest number of exposed pairings, with more coins rewarded for faster guesses. The game was also divided into “stages” to incorporate an element of progression, with the number of new characters increasing by 2 with each stage (e.g., 4, 6, 8, etc.). Additionally, a betting element was added, with participants able to wager based on their confidence in their guess. These changes were well received during follow-up, with participants verbally reporting the game was a “*better format*” and “*much better than last time*.” This was also reflected in the post-modification ratings of the game, with an absolute increase of 30% in the percentage of individuals reporting the game as enjoyable (from 70 to 100%) and an overall increase of 1.3 points in the rating of the game (from 6.3 ± 1.34/10 to 7.6 ± 1.67/10). Importantly, these changes also meant that there was a second game available, in addition to *Farm Quest*, that focused on the training of problem solving/logical deduction.

This positive impact of the changes to the games was reflected in a follow-up survey where, on a scale of 1 (much worse) to 10 (much better), changes to the games were rated 7.91 ± 1.04/10.

Table 6 summarizes the individual and overall rating changes per game post-modification, and Table 7 shows the absolute increase or decrease (in either percentage or point values) from baseline for each individual game, following all modifications.

**TABLE 6:** Individual game feedback, post-modification.

	Content						Usability			Overall rating
	Res.	Enjoyed (% yes)	Enjoyment rating (Av interest + enjoyment)/10	Challenge (% Yes)	Difficulty rating/10)	Features (color/animation/sound)/3	Clear instruction (% yes)	Ease of play (% yes)	Controls/3	
**A Bridge Too Far**	9	100%	7.17 ± 1.58	56%	5.22 ± 1.56	2.59 ± 0.55	89%	89%	2.44 ± 0.53	**8.00** ± **1.58**
Farm Quest	4	100%	8.13 ± 1.34	100%	7.25 ± 0.96	2.83 ± 0.19	50%	50%	2.75 ± 0.50	**7.75 ± 0.96**
Squirrel	5	100%	7.00 ± 1.80	60%	6.20 ± 1.48	2.93 ± 0.26	100%	100%	3.00 ± 0.00	**7.4 ± 1.52**
Snake	4	100%	7.50 ± 0.58	75%	5.75 ± 1.71	2.92 ± 0.29	100%	100%	2.75 ± 0.50	**7.25 ± 0.96**
Sunday Driver	3	100%	7.67 ± 0.58	100%	7.33 ± 1.15	2.56 ± 0.58	67%	100%	2.00 ± 1.00	**7.33 ± 1.15**
Marine Life	4	75%	5.63 ± 3.36	50%	6.67 ± 0.58	2.5 ± 0.58	67%	50%	2.75 ± 0.50	**5.50 ± 3.70**
Swimma	4	67%	6.50 ± 3.00	75%	6.00 ± 2.94	2.67 ± 0.53	67%	75%	2.50 ± 0.58	**6.00 ± 2.71**
Driving Maniac	4	100%	8.38 ± 1.72	100%	5.75 ± 3.40	2.50 ± 0.58	75%	100%	2.50 ± 0.58	**8.50 ± 1.29**
Whack-A-Mole	4	100%	8.63 ± 1.04	100%	7.75 ± 0.96	2.67 ± 0.53	67%	50%	2.75 ± 0.50	**9.00 ± 0.82**
Munchkinis	3	100%	9.33 ± 0.58	100%	6.67 ± 2.31	3.00 ± 0.00	100%	67%	3.00 ± 0.00	**9.33 ± 0.58**
Who’s the Boss?	5	100%	7.20 ± 1.78	80%	6.60 ± 1.82	2.53 ± 0.55	100%	60%	2.60 ± 0.55	**7.60 ± 1.67**
Chow Time	3	100%	9.33 ± 0.58	67%	7.0 ± 1.00	3.00 ± 0.00	100%	100%	3.00 ± 0.00	**9.33 ± 0.58**

*Res, respondents, % indicates percent of participants who answered “yes.” Ratings are on a scale of 1–10 (1 = very poor/easy – 10 = very challenging/enjoyable). Features and controls are on a scale of 1–3 (1 = Not Good, 2 = Average, 3 = Good). For all ratings, the table presents Mean (±SD).*

**TABLE 7:** Absolute increase/decrease from baseline for each individual games following modifications.

	Content					Usability			Overall rating/10
	Enjoyed	Enjoyment rating/10	Challenge	Difficulty rating/10	Features (color/animation/sound/3	Clear instruction	Ease of play	Controls/3	
A Bridge Too Far	+0%	–0.25	–29%	–1.78	–0.22	+20%	–11%	–0.18	**+0.31**
Farm Quest	+64%	+2.4	+0%	–0.11	+0.06	–20%	–5%	+0.05	**+2.25**
Squirrel	+8%	–0.17	–22%	–0.13	+0.2	+0%	+18%	+0.36	**+0.23**
Snake	+58%	+2.96	+0%	–0.92	+0.28	+30%	+67%	+0.93	**+2.42**
Sunday Driver	+33%	+1.23	+0%	–0.11	–0.03	+45%	+78%	–0.62	**+1**
Marine Life	–16%	–1.37	–40%	–0.06	–0.33	–22%	–23%	+0.25	**–1.77**
Swimma	–19%	–0.21	–11%	+0	+0.11	+0.3%	+18%	+0.17	**–0.43**
Driving Maniac	0%	+0.67	+0%	–1.58	–0.02	–11%	+25%	+0.04	**+0.67**
Whack-A-Mole	20%	+1.08	+20%	+0.55	–0.06	–33%	–39%	+0.42	**+1.3**
Munchkinis	+14%	+1.69	+0%	–1.33	+0	+0%	+10%	+0	**+1.76**
Who’s the Boss?	+30%	+1.35	–20%	–0.7	–0.14	–33%	–20%	–0.07	**+1.3**
Chow Time	+20%	+3.03	+37%	+2.8	+0.37	+0%	+0%	+0.25	**+2.93**

*Absolute increase or decrease from baseline is indicated for each measure, presented as either change in percentage (for measures recording % yes) or change in points (for measures using a rating scale). Pre- and post-modification scores can be seen in Tables 4, 6, respectively.*

#### Overall Usability- Post-modification

A post-session survey revealed a positive response to the changes made, with 100% of participants reporting they felt their comments were addressed. Confidence in the ability of repeated use of the NeuroOrb gaming system to be beneficial for cognitive function was reported by all participants (100%) and an overall enjoyment rating of the system was 8.18 ± 1.08/10, which represents an 8% improvement upon the initial system rating in the first session. Importantly, the revised SUS score increased from a 65.58 to a 74.17, which is considered above the average score of 68 on the SUS grading system (Gomes and Ratwani, 2019; Table 5). Using the scale developed by Sauro (2011), this score equates to a grade of “B” (70th percentile) (Sauro, 2011).

## Discussion

This study used a reiterative co-design process to develop a novel serious gaming system, NeuroOrb, for the delivery of CT in individuals with PD. Feasibility was assessed by evaluating a combination of outcomes, including enjoyment, accessibility and acceptability of both the software and hardware. Overall, the NeuroOrb system demonstrated positive feedback in all areas assessed, with integration of feedback resulting in high ratings of enjoyment and confidence in the benefits of the CT program. It is important to note, however, that the lack of a formal motor rating scale, such as the MDS-UPDRS, is a major limitation of the current study, as it is not possible to ascertain from our data whether degree of motor impairment affected participants’ perceptions of either the usability or enjoyability of the NeuroOrb, which could have impacted upon system ratings.

The cohort in the current study was high functioning, with no evidence of CI (average MMSE = 29), depression or motor impairment significant enough to interfere with daily activities. This is surprising, as the cohort included a broad range of disease duration, ranging from 1 to 19 years (average 8 years ± 5.43). Given that participants in the co-design trial were not specifically recruited based on cognitive function (i.e., PD without CI, PD-MCI, and PD-D), this may represent a selection bias, with only high functioning individuals volunteering to take place in the study. This may make it difficult to interpret how those with PD-MCI or PD-D would have engaged with the NeuroOrb system and whether they would have been able to understand the game instructions, even post-modification. Additionally, given the lack of sensitivity of the MMSE to detect either MCI or dementia in PD (Hoops et al., 2009), the group may have been more impaired than suggested based on MMSE scores alone. In support of this, a percentage of the participants self-reported cognitive difficulty [remembering events (30.1%), remembering information (38.5%), paying attention (15.4%), learning new tasks (15.4%), remembering words (15.4%), and managing day-to-day tasks (15.4%)]. It is possible that a measure more sensitive to detecting CI in PD, such as the Montreal Cognitive Assessment (MoCA) (Hoops et al., 2009), may have been better able to detect some cases of MCI or mild dementia in the current cohort. Nevertheless, while it may be difficult to extrapolate the results from this small sample to the wider PD population, including those with more severe motor and CIs, this is also likely to represent the target demographic who may derive the most benefit from CT. In support of this, recent research supports that older adults with higher baseline cognitive function are more likely to benefit from CT than those who are already impaired (the so-called magnification effect) (Mohlman et al., 2011; Fu et al., 2020).

Encouragingly, initial impressions of the NeuroOrb system were positive, with the majority of participants reporting that they enjoyed the system and rating it highly. Indications of acceptability for the implementation of the CT program were also high, with most participants expressing confidence in the NeuroOrb system to improve or maintain cognitive function. These are important positive predictors, as game enjoyment and perceptions of cognitive benefit toward gamified CT in an older population have been correlated with motivation (Boot et al., 2016). Accordingly, high levels of initial enjoyment and confidence reported in the NeuroOrb system are likely to reflect motivation to engage further with the system. In support of this, 10 out of 13 of the participants in the current study reported that they would use the NeuroOrb system several times per week, or even daily, if it were available commercially. This is particularly relevant in PD, where ∼40% of individuals experience disorders of motivation (den Brok et al., 2015). Such motivation to engage may, in turn, translate to improvements in adherence to a long-term CT regime, enhancing the efficacy of the CT program overall.

Concerningly, the Orby controller itself received only a moderate rating for accessibility after the first exposure to the system. Given the challenges associated with motor function in the PD population (Mazzoni et al., 2012), it is important for the controller to be considered accessible, and initial accessibility feedback was not as positive as hoped. This rating could be related to several factors; for example, it may indicate issues with handling of the controller sensitivity and responsiveness of the controller to specific games, or ergonomics of the overall set-up. Furthermore, this rating may be reflective of the minimal exposure and unfamiliarity of the participants to computerized games, specifically video games. Minimal experience with handling similar technologies suggests a steeper learning curve, which may also have impacted initial accessibility impressions of the controller. Over time, participants appeared to become more comfortable with controller use and were able to optimize their own use of the Orby controller. This versatility exemplifies a positive adaptive feature of the Orby controller, allowing it to cater for the heterogeneity of motor impairments in the PD population (Greenland et al., 2019).

Although individuals did become more comfortable with the system with repeated exposure, however, some problems persisted, with an overall below-average SUS indicating compromised usability. While unclear exactly what may have driven these difficulties, they may have been due to either general setup/handling issues or to participant fatigue/discomfort. Fatigue is a commonly reported symptom in PD, with a reported prevalence of 50% (Siciliano et al., 2018) and is one of the three Rs (i.e., “Repeatedly”) suggested by Kouroupetroglou (2014) to represent particular barriers for computer use in motor impaired populations. As such, it may have affected the ability of participants to engage throughout the session and, in turn, affected their perception of the usability of the system overall. It is also possible, however, that difficulties with the games themselves negatively impacted on overall system usability, making it critical to interpret overall usability in the context of ratings of the gaming suite.

Overall, the response to the games was positive, with all games (except *Snake*) receiving an above average overall rating. In terms of enjoyment, the majority of games were considered enjoyable. This is important, as enjoyment is a strong motivator and positively associated with effortful engagement (Cacioppo et al., 1996). Games with a below average (<50%) number of participants reporting enjoyment included *Farm Quest* and *Snake*. Lower participant enjoyment of these games may have been reflective of a number of factors, such as inappropriate game difficulty or issues with game features.

The relationship between perceived task difficulty and performance is not well understood for CT in PD; however, studies suggest a balance is important. In older adults specifically, Selective Engagement Theory (Hess, 2014) proposes that increased ‘cognitive costs’ associated with activities later in life results in a reduction in the cost/benefit ratio, reducing the willingness of older adults to engage in demanding activities (Hess et al., 2016). It is critical to balance this, however, against the theoretical framework proposed by Lövdén et al. (2010) for achieving cognitive plasticity in adults. According to this model, the transfer of gains from CT across multiple cognitive domains or to real-world contexts depends on the difficulty of the training task, with sustained cognitive challenges required to induce lasting neural changes. This necessitates a continual mismatch between the demand of the task (i.e., the cognitive load) and the cognitive capacity of the individual. In support of this, adaptive training of working memory (i.e., where task demands are continually increased based on performance) resulted in far transfer to an untrained episodic memory task, as well as accompanying neural changes (Flegal et al., 2019). In light of these considerations, we sought to achieve an effective challenge, while also reducing “cognitive cost.” While we were able to achieve this for the majority of games, *Chow Time!* was rated as too easy and *Munchkinis* was rated as too difficult. Interestingly, however, this did not appear to affect enjoyment of these games.

Similarly, enjoyment of the games did not appear to be negatively affected by game features, with use of color, animation and sound all highly rated. This is important, as basic stimuli (images and texts) associated with traditional pen and paper CT can make therapy boring for patients (Alloni et al., 2017). The inclusion of 3D graphics in computerized training is considered beneficial, due to increased entertainment and involvement of the patient, as well as the introduction of new elements (such as spatial perception) into training, ultimately improving direct interaction compared to more abstract 2D counterparts (Alloni et al., 2017). Given that neither game difficulty or features appeared to negatively impact either enjoyment or rating of the game, it may be that usability of the games themselves, including clarity of instructions provided and controller responsiveness for each game, was most important for determining the enjoyability and overall rating of the game. In support of this, both *Farm Quest* and *Snake*, the two lowest rated games for enjoyment, scored poorly for ease of play, with both also receiving only moderate ratings for clarity of instructions.

Encouragingly, through the co-design process and the subsequent alterations made to the NeuroOrb system, we seemed to successfully address all points raised, with 100% of respondents stating that they felt as though their feedback was addressed. Following implementation of these changes, participants rated changes to both the controller and the games extremely highly. Furthermore, overall enjoyment of the system as a whole increased and there was a notable improvement in SUS score from the 42nd to the 70th percentile (Sauro, 2011), indicating that NeuroOrb is likely to have high usability as a tool for individuals with PD.

Importantly, the number of issues that we were able to pre-emptively identify and address through this study highlights the importance of engaging in a co-design process with key stakeholders, prior to engaging in a large-scale intervention trial. Such a cooperative approach is in line with current best practice guidelines for the design of interventions for use in patient populations, and is anecdotally reported to lead to more effective services and better outcomes for individuals (although more rigorous assessment of outcomes and cost-benefit analysis is needed) (Clarke et al., 2017). Co-design has previously been successfully used in many healthcare indications. In PD specifically, co-design has been used to design eHealth services (Revenas et al., 2018), collaborative care (Kessler et al., 2019), and even smart home technology (Bourazeri and Stumpf, 2018). Most recently, and highly relevant to the current work, co-design was used to put forth recommendations for the design of a personalized gaming suite for use by individuals with PD (Dias et al., 2020). Within the current study, without consultation with key stakeholders in a reiterative co-design process, many of the issues we identified would have been missed. This could have had a disastrous impact on any intervention trials, as issues with the games themselves (e.g., understanding what the objective is) or with the hardware (e.g., navigating the controller or avoiding fatigue) could have negatively affected engagement with the system, or even successful completion of the trial. This, in turn, may have confounded the assessment of effects on cognitive function, potentially masking any benefits derived from NeuroOrb. Instead, following our extensive incorporation and evaluation of suggested modifications, we are now well-placed to proceed to a large-scale clinical trial using NeuroOrb to deliver customized CT in individuals with PD, evaluating any potential benefits using a sensitive and comprehensive cognitive assessment battery.

## Conclusion

Considering the positive ratings of the controller, gaming suite and the NeuroOrb system overall, we have developed a customized “Serious Games” approach to CT, optimized for use in individuals with PD and ready for deployment in subsequent intervention trials designed to assess its efficacy. Through our co-design process, we believe that the incorporation of novel elements into both the hardware and software of the NeuroOrb system represent a significant improvement on other CT systems developed to date, which often use commercially available software packages or non-validated paradigms, without consultation from key stakeholders (Thompson et al., 2020). Instead, our system allows us to target the areas of cognitive function that present the most concern for individuals with PD in an accessible and highly engaging way. This makes us well-placed to obtain maximal benefit from use of the system to deliver CT in this population. While it ultimately remains to be determined if NeuroOrb will result in cognitive benefits for individuals with PD, or whether such benefits will last or transfer to everyday ADLs, this process nevertheless illustrates the importance of co-design and appropriate consultation of key stakeholders when designing future therapeutic strategies.

## Data Availability Statement

The raw data supporting the conclusions of this article will be made available by the authors, without undue reservation.

## Ethics Statement

The studies involving human participants were reviewed and approved by Human Research Ethics Committee of the University of Adelaide (H-2020-214). The patients/participants provided their written informed consent to participate in this study.

## Author Contributions

BG coordinated the co-design trial, analyzed the data, and drafted the manuscript. DH designed the NeuroOrb system, contributed to the co-design trial, and supervised the project. BW was the game developer for the software suite. BE, AM, and SD contributed to the co-design trial and analyzed the data. LC-P contributed to the design of the software suite, led the co-design trial, conducted data analysis, supervised the project and substantially revised the manuscript. All authors contributed to the final version of the manuscript.

## Conflict of Interest

The authors declare that the research was conducted in the absence of any commercial or financial relationships that could be construed as a potential conflict of interest.

## Publisher’s Note

All claims expressed in this article are solely those of the authors and do not necessarily represent those of their affiliated organizations, or those of the publisher, the editors and the reviewers. Any product that may be evaluated in this article, or claim that may be made by its manufacturer, is not guaranteed or endorsed by the publisher.
